# NEWHINTS cluster randomised trial to evaluate the impact on neonatal mortality in rural Ghana of routine home visits to provide a package of essential newborn care interventions in the third trimester of pregnancy and the first week of life: trial protocol

**DOI:** 10.1186/1745-6215-11-58

**Published:** 2010-05-17

**Authors:** Betty R Kirkwood, Alexander Manu, Charlotte Tawiah-Agyemang, Guus ten Asbroek, Thomas Gyan, Benedict Weobong, R Eric Lewandowski, Seyi Soremekun, Samuel Danso, Catherine Pitt, Kara Hanson, Seth Owusu-Agyei, Zelee Hill

**Affiliations:** 1 Department of Nutrition and Public Health Intervention Research, London School of Hygiene and Tropical Medicine, Keppel Street, London WC1E 7HT, UK; 2 Kintampo Health Research Centre, Ghana Health Service, PO Box 200, Kintampo, Brong Ahafo Region, Ghana; 3 Department of Counseling and Clinical Psychology, Columbia University, New York, USA; 4 Department of Global Health and Development, London School of Hygiene and Tropical Medicine, Keppel Street, London WC1E 7HT, UK; 5 Institute of Child Health, University College London, 30 Guilford Street, London WC1N 1EH, UK

## Abstract

**Background:**

Tackling neonatal mortality is essential for the achievement of the child survival millennium development goal. There are just under 4 million neonatal deaths, accounting for 38% of the 10.8 million deaths among children younger than 5 years of age taking place each year; 99% of these occur in low- and middle-income countries where a large proportion of births take place at home, and where postnatal care for mothers and neonates is either not available or is of poor quality. WHO and UNICEF have issued a joint statement calling for governments to implement "Home visits for the newborn child: a strategy to improve survival", following several studies in South Asia which achieved substantial reductions in neonatal mortality through community-based approaches. However, their feasibility and effectiveness have not yet been evaluated in Africa. The Newhints study aims to do this in Ghana and to develop a feasible and sustainable community-based approach to improve newborn care practices, and by so doing improve neonatal survival.

**Methods:**

Newhints is an integrated intervention package based on extensive formative research, and developed in close collaboration with seven District Health Management Teams (DHMTs) in Brong Ahafo Region. The core component is training the existing community based surveillance volunteers (CBSVs) to identify pregnant women and to conduct two home visits during pregnancy and three in the first week of life to address essential care practices, and to assess and refer very low birth weight and sick babies. CBSVs are supported by a set of materials, regular supervisory visits, incentives, sensitisation activities with TBAs, health facility staff and communities, and providing training for essential newborn care in health facilities.

Newhints is being evaluated through a cluster randomised controlled trial, and intention to treat analyses. The clusters are 98 supervisory zones; 49 have been randomised for implementation of the Newhints intervention, with the other 49 acting as controls. Data on neonatal mortality and care practices will be collected from approximately 15,000 babies through surveillance of women of child-bearing age in the 7 districts. Detailed process, cost and cost-effectiveness evaluations are also being carried out.

**Trial registration:**

http://www.clinicaltrials.gov (identifier NCT00623337)

## Background

Although the child survival revolution of the 1980s led to dramatic reductions in overall child mortality, it has had little impact on deaths taking place in the first 28 days of life (the neonatal period). There are just under 4 million neonatal deaths, accounting for 38% of the 10.8 million deaths among children younger than 5 years of age taking place each year[1]; 99% of these occur in low- and middle-income countries[1]. Tackling neonatal mortality is therefore essential if the millennium development goal of reducing child mortality by two-thirds between 1990 and 2015 is to be achieved[1].

Common direct causes of neonatal deaths in developing countries are known: infections (pneumonia, neonatal tetanus, sepsis, and diarrhoea), asphyxia, birth injuries and complications of preterm birth[1]. Indirect causes of neonatal deaths such as low birth weight and hypothermia are also important [2] as is the link between maternal health and neonatal outcomes [1,2]. Postnatal care for mothers and neonates in developing countries, particularly when deliveries occur at home, is either not available or is of poor quality. Interventions are urgently needed, particularly those directed at improving family newborn care practices and community level health service delivery; the Lancet neonatal series suggests that 15-32% of neonatal deaths could be prevented through promotion of a few key practices: clean home delivery, hygienic cord care, thermal care, early and exclusive breastfeeding and care seeking for illness[3].

Trained community workers are considered by many to be pivotal to newborn care in the community, as they can act as catalysts for community actions and also be providers of care[4], and several studies in South Asia have shown that substantial mortality reductions can be achieved with this approach[5-8]. Projects in Nepal[9] and Bolivia[10] have demonstrated that substantial improvements in neonatal survival can also be achieved through encouraging community organisation and participation in women's groups.

Based on the successes from the studies in South Asia, WHO and UNICEF have issued a joint statement calling for governments to implement "Home visits for the newborn child: a strategy to improve survival" [4]. However, the feasibility and effectiveness of community approaches to reduce newborn mortality have not yet been evaluated in Africa, where the epidemiology of neonatal deaths and the health system are very different from South Asia. Progress in reducing neonatal mortality has been slower in Sub-Saharan Africa than in any other region in the world, and projections on percentage of skilled attendance at delivery suggest that this will remain static at just above 40% over the period to 2015[11]. Complementary strategies, such as delivering community-based interventions, are urgently required[3]. This paper presents the protocol for a cluster randomised controlled trial to evaluate the impact of such a community-based intervention on newborn care practices and neonatal mortality in rural Ghana. This is called Newhints: NEWborn Health INTervention Study

## Methods

### Aim

To develop a feasible and sustainable community-based approach in rural Ghana to improve newborn care practices and careseeking during pregnancy and childbirth, and by so doing improve neonatal survival.

### Primary objectives

1. To link with District Health Management Teams (DHMTs) to develop a feasible and sustainable intervention to improve newborn care practices and careseeking through training the current network of community based surveillance volunteers (CBSVs) to identify pregnant women in the community and to conduct two home visits during pregnancy and three in the first week of life of the neonate.

2. To evaluate the impact of these home visits on all cause neonatal mortality.

3. To evaluate their impact on newborn care practices.

### Secondary objectives

4. To assess the coverage and quality of the service provided and the family and community response to the service.

5. To assess the cost of implementing the intervention, and the cost-effectiveness of any impact.

6. To evaluate whether the impact of the intervention on neonatal mortality differs between home- and facility-based deliveries.

7. To evaluate the impact of the intervention on age- and cause-specific neonatal mortality.

### Setting

The Newhints trial is part of a long-term collaboration between the London School of Hygiene & Tropical Medicine, the Institute of Child Health and the Kintampo Health Research Centre (KHRC) in the Ghana Health Service. Newhints is based at KHRC and covers seven contiguous districts in the Brong Ahafo Region of Ghana: Kintampo North, Kintampo South, Wenchi, Tain, Techiman, Nkoranza North, and Nkoranza South. These districts also formed the study area for the vitamin A and maternal mortality "ObaapaVitA" trial. More than 15,000 babies are born within this area each year; the neonatal mortality rate is 31 per 1000 live births and approximately 50% of births occur at home[12].

The study area lies within the forest-savannah transitional ecological zone, and has two distinct rainy seasons from April to July and from September to October. The area is densely populated (175 people/square mile) with a total population of approximately 600,000 persons, and more than 100,000 women of reproductive age. The annual population growth rate is currently 3.1%; only 10% of the population in the study area live in the urban district administrative centres. The rural population lives in compounds, containing houses with mud walls, and thatch or aluminium roofs, in dispersed villages surrounded by farming land. The main occupation is subsistence farming and the main crops are yam, maize and millet. The population is multi-ethnic and education levels are low.

There are 4 district hospitals (3 hospitals are currently shared by two districts) that provide clinical (outpatient and inpatient) and maternity services and act as the first referral point for sub-district and community based health care facilities. The sub-district has an administrative centre located in a small town and usually has a health centre that provides basic maternal and child health (MCH) care. At community level there are a small number of additional government health centres and private facilities that provide basic MCH services. Each village also usually has one or more traditional birth attendants (TBAs), trained or untrained, one or more community-based surveillance volunteers (CBSVs) who assist the DHMT with registration of births, mobilisation of the community for activities such as national immunisation days, registration of deaths, and with community child welfare outreach clinics. Other community based health care providers are chemists/drug sellers and traditional healers.

### Overview of Trial Design

The Newhints intervention is being evaluated through a cluster randomised controlled trial design. The clusters are Newhints zones which correspond to supervisory units of about 8-12 CBSVs. There are 98 Newhints zones in total; 49 zones randomised for implementation of the Newhints intervention, with the other 49 zones acting as controls. The trial planning started in October 2006. The Newhints intervention was developed and fully implemented in the intervention zones by the end of 2008. Impact data on neonatal mortality and newborn care practices is being collected through ongoing surveillance of all women of child-bearing age and their infants in the trial area, and will be based on approximately 15,000 babies born from 1 January 2009. Detailed process, cost and cost-effectiveness evaluations are also being carried out. Data collection is expected to be completed in April 2010 and analysis will take place throughout 2010.

### The Newhints Intervention

**Figure 1:**
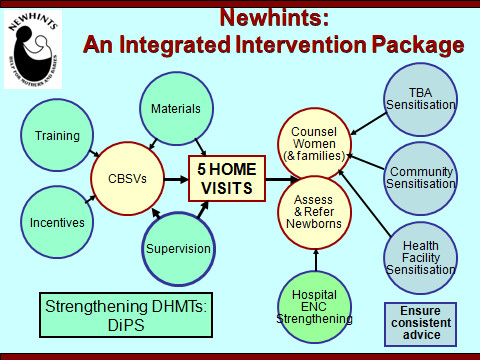
**Newhints integrated intervention package details**.

Newhints is an integrated intervention package (Figure 1) based on extensive formative research [13], and developed in close collaboration with the District Health Management Teams (DHMTs), with input from key national neonatal policy makers and programme coordinators, and experts in neonatal health, behaviour change communication and working with community volunteers.

#### DHMT collaboration

The Newhints intervention was developed in close collaboration with the DHMTs. Each DHMT designated a member to be the liaison person for all Newhints-related activities, to attend regular DHMT-Newhints meetings involving all districts in the trial area, and to take a lead role in introducing the Newhints intervention to the health facilities and communities. The DHMTs receive a small quarterly budget to cover costs of their participation. In addition, there are 2 district project supervisors (DiPS) based in each DHMT; they participate in other community DHMT activities, such as the national immunization campaigns, as well as supervising the CBSVs. All DiPS are provided with a motorbike and fuel and maintenance costs are covered by the project.

#### Home visits by CBSVs

The core component of the intervention is five home visits by CBSVs to pregnant women and their babies. Two visits are targeted during pregnancy and three during the first week of life of the neonate; the timing and focus of each visit is summarised in Table 1. The visits involve family members as well as the pregnant woman and use storytelling and a counselling and problem solving approach concerning key gaps in care practices identified during the formative research. At the first visit after birth, the CBSV weighs the baby, and advises mothers of low birthweight (LBW) babies (<2500 g) about a package of special care comprising skin to skin contact, frequent breastfeeding, wiping rather than bathing the baby, and special attention to hygiene. The CBSVs also refer any very LBW babies (<1500 g) to hospital. In addition, the CBSVs assess all babies at each of the three postnatal visits and refer to hospital any baby who has one or more of the following danger signs: not able to feed since birth or stopped feeding well; convulsed or fitted since birth; fast breathing: two counts of 60 breaths or more in one minute; chest in-drawing; high temperature: 37.5°C or more; very low temperature: 35.4°C or less; only moves when stimulated; yellow soles; pus from umbilical stump or red umbilical stump; pus from eyes; and boils with pus. They conduct follow-up visits for referred babies within 24 hours, and an additional postnatal visit to LBW babies at the end of the second week.

**Table 1:** Newhints visit schedule and content.

Early pregnancy	Key messages:
	- Promote and plan for a facility delivery
	- Plan for a clean home delivery
	- Plan for emergencies
	- Sleep under a treated bed net
	
	Supporting messages:
	- Encourage antenatal care attendance
	- Seek care for maternal danger signs
**3^rd ^trimester**	- Dry, wrap & breastfeed immediately after delivery (plus 2nd assistant during home delivery to facilitate this)
	- Delay bathing for at least a day

**Day of birth**	- Weigh and assess the baby for danger signs
	- Refer very low birth weight (LBW) & potentially sick babies to hospital
	- Encourage exclusive breastfeeding (EBF)
	- Encourage good thermal care (bath with warm water, dry immediately and wrap well)
	- Encourage special care for LBW babies (Skin to skin contact, delay bathing at least 3 days, hygiene, frequent breastfeeding)

**Day 3**	- Assess baby for danger signs & refer sick babies
	- Reinforce EBF, thermal care
	- Teach newborn danger signs & encourage prompt care-seeking

**Day 7**	- Assess baby for danger signs & refer sick babies
	- Reinforce EBF, thermal care, prompt care-seeking
	- Encourage bed net use, immunisations

**Other visits**	- Follow-up visits within 24 hours for referred babies
	- Visit at 14 days for LBW babies

#### CBSV Materials and Equipment

CBSVs are provided with a set of materials that aim to motivate and give credibility as well as serving functional roles. These are: picture ID; waterproof Newhints bag; Newhints polo shirt; manual; workbook; counselling and assessment cards; tubular weighing scales and slings; digital timers to measure respiratory rates; digital thermometers; cotton rolls and 70% ethanol for disinfecting thermometer; referral slips; and family cards to record appointments, births, birthweights and referrals and which also have key message reminders. CBSVs, who work in areas that are too large to be covered easily on foot, are provided with a bicycle.

#### Training of CBSVs

It was decided that this complex intervention would be best introduced to the CBSVs in two phases of training with phase 1 focusing on identifying pregnant and delivered women in the community, behaviour change communication, essential newborn care and on the use of counselling cards. Three-day training courses with 30-40 CBSVs per course took place during February and March 2008 in locations accessible to the CBSVs such as schools, churches and health facilities with CBSVs travelling to the training venue each day for the 3-day course. Training was led by teams of 2-4 Newhints staff, who had attended a training of trainers (ToT) session conducted by the Newhints clinician (AM), who had himself attended a UNICEF run training of trainers course. It utilized a competency based approach. Facilitator and participant guides and a set of overheads were developed by adapting various WHO, UNICEF and SNL manuals.

Phase 2 training focussed on weighing, assessing the newborn for danger signs and referring, and on promoting special care for low birthweight babies; it also included a review of phase 1 activities. It started with a ToT workshop conducted by Dr Rajiv Bahl (WHO) in Accra in May 2008 for eight Newhints trainers. The content of the training package was finalised during the ToT to be delivered over four days with a maximum of 25 participants per session, and involving practical sessions where CBSVs could practise weighing and assessing newborns. The Newhints district project supervisors (DiPS) were trained at the end of May 2008. The second phase of training for the CBSVs started on June 2, and was completed on 12 July 2008. In addition, CBSVs received a 2 day refresher training course at the end of October and beginning of November 2008. A total of 406 CBSVs were fully-trained, with all intervention communities having one or more trained CBSVs.

#### Supervision of CBSVs

There are two District Project Supervisors (DiPS) based in each DHMT, who have been trained in supervisory skills and who are responsible for supervising CBSVs in their catchment areas. They aim to visit each of their CBSVs at least once a month to directly observe a home visit and to problem-solve any issues. They also aim to hold group meetings every two to three months where CBSVs can share their experiences and problems are discussed. In addition, they hold meetings with community leaders to provide feedback and stimulate interest in the intervention. They also carry out regular checks on all CBSV equipment and arrange replacements as necessary. A set of materials have been developed to support supervision including a workbook to record activities and issues raised, a monthly CBSV tally sheet to record visits carried out and participation in group meetings, and forms to record detailed observation of home visits.

#### Incentives

It was decided during the formative research that providing a monthly monetary incentive would be key in keeping CBSVs active and maintaining motivation. An amount of 5 Ghana cedis per month (approximately $5) was determined in discussion with national and district level representatives of the Ghana Health Service to be both sustainable and sufficient to motivate CBSVs. These monthly incentives are distributed by the DiPS during supervisory visits.

#### Hospital essential newborn care strengthening

As CBSVs are trained to refer very low birthweight and sick babies, and as the formative research identified some inadequacies in the current provision of newborn care, it was considered essential to update skills and knowledge of staff in the main health facilities. In response to a joint request from the DHMTs and Newhints team, endorsed by the National Reproductive and Child Health Coordinator, WHO conducted a national ToT workshop in "Strengthening Essential Newborn Care in Health Facilities" in Accra in July 2008. Two training workshops were then held later in July at Techiman and at Nkoranza hospitals for staff from the 10 largest health facilities, including the district hospitals, that provide care and services for newborns; these were facilitated by the Newhints clinician (AM) and others trained at the national workshop.

#### Supportive activities

There are several supportive actions to promote the intervention and ensure women receive consistent advice from health facility staff, traditional birth attendants (TBAs) and other community members. Each of these has their own protocol.

♦ ***Health facility sensitization: ***The DHMTs organised meetings in each district during September and October 2007 and invited sub-district teams, and those in charge of health facilities together with the public health nurses and midwives who help in the delivery of babies and who take care of pregnant mothers. At these meetings, the Newhints team outlined the proposed intervention and discussed its implications for the health facilities. This included a detailed discussion of the newborn care practices being promoted in order to harmonise messages between the trial and health facility staff, and feedback on findings of the health facility survey conducted during the formative research. In addition, the six district hospitals were visited in June and July 2008 in order to refresh the memory of health workers in the hospital regarding Newhints, to introduce and explain the referral strategy and the use of the referral slip, and to discuss prioritization of babies with referral slips.

♦ ***Community leaders sensitization: ***Introductory visits were made during December 2007 and January 2008 to all 191 communities in the intervention zones by teams of one DHMT representative, one Newhints supervisor (DiPS) and one NewHints researcher. Appointments were made with community leaders, who invited key members of their community; the CBSVs also attended. The meetings aimed to garner community leader support for Newhints activities, and to raise the profile of the CBSVs. They lasted 1 to 2 hours and took the form of presentation, demonstration and discussion. The questions that were raised centred on issues around implementation, community involvement, financial support and the content of Newhints intervention messages. These fed into the CBSV training manual and the TBA sensitization and community-wide meeting (durbar) protocols. The community leaders were formally asked if they would like their CBSVs to carry out Newhints activities; all agreed.

♦ ***TBA sensitization: ***A series of TBA sensitisation meetings were held in February 2008 in each district to garner their support for Newhints activities, to help ensure that TBA advice would not conflict with Newhints advice, and to discuss behaviours that TBAs may control such as hand washing, early bathing and immediate drying and wrapping. All TBAs (trained and untrained) who were known to be active within the intervention communities were invited.

♦ ***Community durbars: ***Community wide meetings were organised by the DHMT-Newhints teams during July and August 2008, and chaired by the community chiefs. Their purpose was to introduce the importance of newborn care to the community, to explain the rationale, content and structure of the Newhints intervention, to discuss the importance of community support for its success, and to present the fully trained CBSVs with their Newhints T-shirt, bag and certificate.

### Mapping zones

An inventory was carried out of all CBSVs working in the trial area and data collected on their socio-economic status, level of education, and current workload and schedule. The trial area was then divided into a total of 98 supervisory zones. Their boundaries were defined in discussion with the DHMTs, based on feasibility of coverage within the zone by bicycle, size of communities, geographical access from one community to another, and the total number of CBSVs covered aiming for about 8 CBSVs per zone. There were a few larger zones as villages were never divided between zones and some had more than 8 resident CBSVs, and a few smaller ones in geographically separated communities. The large towns were divided into zones of geographical non-contiguous areas, based on size, population and already established CBSV work areas.

### Randomisation

Meetings were held in each district in November 2007 to introduce the Newhints trial to all CBSVs, to explain the proposed randomization process and to obtain their cooperation and support for this; 686 CBSVs (91%) attended. Forty-nine zones were then selected at random for implementation of the Newhints intervention, with the other 49 zones acting as control. This was carried out by an independent epidemiologist using restricted stratified randomisation to ensure balanced numbers of intervention and control zones in each of 10 strata. These were the four large towns (Kintampo, Nkoranza, Techiman and Wenchi) and the six districts (Kintampo North, Kintampo South, Nkoranza, Tain, Techiman and Wenchi) minus these towns; note this took place before Nkoranza was divided into two districts, Nkoranza North and Nkoranza South. Restricted randomisation used available surveillance data to ensure that intervention and control arms were also balanced with respect to the following criteria: absolute differences of less than 2/1000 live births for neonatal mortality rates, less than 2.5% for the percentage of deliveries in a health facility, and less than 2.5% for the percentage of deliveries in a private hospital, in each of 2004, 2005 and 2006. An additional selection criterion was to ensure that the 4 pilot zones (which had been chosen at random) were allocated to the intervention group.

### Intervention Zones

The Newhints intervention as described above was implemented in the 49 intervention zones. All pregnant women and newborns living in these zones were therefore potential recipients of the intervention receiving home visits from CBSVs, in addition to routine maternal and child health (MCH) care currently available.

### Control Zones

Pregnant women and newborns living in the control zones continued to benefit from the routine MCH care currently available, which includes: antenatal clinics (ANC), Infant Welfare Clinics (IWC), access to free delivery with skilled attendants, access to TBA delivery and care, and routine interactions with CBSVs concerning outreach MCH and immunisation clinics. In addition control zones benefitted from the hospital essential newborn care strengthening and health facility sensitisation that covered all facilities in the trial area.

### Sample size

The sample size was determined by the primary outcome, all cause neonatal mortality, using the baseline neonatal mortality rate (NMR) of 31 per 1000 live births and the intraclass correlation coefficient (ICC) of 0.0007256, where the ICC[14] is defined as the ratio of the between zone variation to the total variation. This suggests that a total sample size of 15,200 livebirths would have 80% power to detect a 25% reduction in NMR at the 5% significance level, 93% power to detect a 30% reduction and 60% power to detect a 20% reduction. This sample size should be achieved by the number of livebirths that take place in a year in the trial area. The evaluation will be based on data collected for all babies born from 1 January 2009; this is 1 month after the intervention was fully implemented, and 6 months after CBSVs started assessing babies in July 2008 as well as counselling about newborn care practices.

### Impact evaluation

The primary outcomes are all cause neonatal mortality and key care practices; these will be compared between intervention and control zones. Secondary outcomes are age and cause-specific neonatal mortality. All required data are being collected through the surveillance system of 4-weekly home visits to all women of reproductive age established for the ObaapaVitA vitamin A and maternal mortality trial that took place from December 2000-October 2008[15]. This surveillance has been continued for the Newhints trial.

Resident fieldworkers are responsible for a fieldwork area (FWA) of four contiguous clusters of compounds, visiting women in one cluster per week over a 4-weekly cycle. Each week, fieldworkers receive an updated listing of women to be visited that week, and their pregnancy status, arranged by compound. A MONTH form is completed for each woman, and includes questions on whether she was present, if not whether she had died, any morbidity requiring treatment outside the home or hospitalisation, her pregnancy status, and a question on the outcome of the pregnancy, completed when a pregnancy ends. There is a scheduled 4-week fieldwork break each year over Christmas.

Other forms are completed as required. A PROFILE form collecting socio-demographic information is completed as soon as a woman reports that she is pregnant. A birth results in: a BIRTH form collecting data on pregnancy, delivery, the baby (or babies), newborn care practices and contact with CBSVs; and monthly INFANT form(s) completed until the baby reaches 12 months of age collecting data on their status, and exposure to key child survival interventions. These forms were revised to ensure that they capture data on practices promoted by the Newhints intervention.

Verbal post-mortems (VPMs) are carried out for all neonatal deaths in the trial area. A surveillance supervisor visits the household and interviews the mother or care-taker about the circumstances surrounding the death, including an open history, and specific questions on symptoms. All VPMs are reviewed by two experienced doctors, who independently code the likely cause of death. If they disagree, the form is reviewed by a third doctor; if their diagnosis matches one of the other two, this is accepted. If not, they meet to discuss the case and attempt to reach agreement. If this is not possible the cause is coded as unable to be determined.

#### Data Management

The trial impact evaluation outcomes will be derived from the surveillance database which was established in 2000 using Visual FoxPro (version 6.0 Microsoft Corp Seattle WA USA), and which was modified to include new data collection forms developed for Newhints. All forms are manually checked for completeness and consistency before they leave the field, collected and processed on a weekly basis. Independent double data entry with verification is carried out together with range and consistency checks, and inter-table consistency checks. Any queries identified are resolved promptly by the trial management team, and the database updated. New data are added to the database within 4 weeks of collection, and in time for the updated data to be used to generate field listings for the next 4-weekly visit. Copies of the surveillance database will be made and frozen within three months after the completion of the fieldwork.

#### Participant flow & comparability of treatment arms

A flow diagram will be completed showing the number of zones, pregnancies, livebirths, neonatal deaths and loss to follow-up in the intervention and control arms, together with a map showing the locations of the intervention and control zones. Intervention and control zones will be compared with respect to the following variables: neonatal mortality rate, the percentage of skilled attendants at delivery and percentage of deliveries occurring in health facilities in 2007 (baseline); level of education of mothers, their ethnic group of origin, marital status and parity, and occupation (used as proxy indicator for the level of income), since these are known either to be related to the neonatal mortality rate or to effect peoples' knowledge, attitudes and practices on neonatal care. No statistical significance tests will be carried out on these comparisons [14,16]. However, analyses will be carried out both including and excluding these potential *a priori *confounders.

#### Intention-to-treat analyses

The primary analysis for each outcome will be intention-to-treat, where intention to treat is defined by a woman's zone of residence. All analyses will account for the cluster-randomised design using random effects logistic regression and will be carried out both with and without adjustment for potential confounders (see above); individual-level methods are statistically more efficient than cluster-level methods, and are preferred when a large number of clusters have been randomised, as is the case in this trial, as they readily allow adjustment for covariates [16]. Quadrature checks will be carried out to confirm the reliability of the results; should these fail generalized estimating equations (GEE) and robust standard errors will be used instead [16]. The estimated effect of the intervention will be presented as a relative risk together with a 95% confidence interval. The intraclass correlation (ICC) and coefficient of variation (k) will be reported. Random-effects logistic regression will also be used to explore whether there are any differences in impact of the intervention: between facility- and home-based deliveries; between urban and rural areas; and between the four zones included in the pilot and the other intervention zones.

#### Secondary analyses

With a public health intervention, such as Newhints, it is impossible to ensure every eligible recipient receives the intervention in exactly the way it was intended. Thus it is likely that only a proportion of pregnant women residing in intervention zones will receive all five home visits at the timing intended; others may not have received any, or fewer visits, or visits later than intended, in particular the first post-natal visit may not have taken place within 24 hours as intended. Secondary analyses will therefore also be carried out to examine whether the impact of the intervention varies according to the number and timing of home visits each woman has received, and the average quality of the intervention delivered in the zone, as assessed by the process indicators measured on a sub-sample of women (see below). This will be explored both using individual quality indicators and by dividing intervention zones into quintiles, based on a quality index derived using principle component analysis [17].

### Process evaluation and intervention monitoring

All aspects of the intervention process are being fully documented and evaluated on an ongoing basis using a variety of methods and data sources:

♦ *CBSV Programme: *The CBSV database will provide data on the following: Profile of the CBSVs (age, gender, ethnicity); Number (& %) of CBSVs trained, & retrained; CBSV attrition and replacement rate; Number (& %) of CBSVs who received incentive payment each month. This will be supplemented by in-depth interviews with a sample of CBSVs, and issues raised during group meetings.

♦ *Supervisor performance: *This is being assessed on an ongoing basis using data collected from the DiPS workbooks, monthly log sheets and observations of supervisory visits by their supervisors. Indicators include: % CBSVs who received supervisory visits each month; % CBSVs who were directly observed during supervisory visits each month; % of CBSVs who attended group meetings in each 2 monthly period; frequency of supervisory visits per CBSV; Frequency of group meetings per CBSV. In addition supervisor performance will be assessed by % supervisory visits observed by a senior newhints team member that were conducted according to protocol; and % supervisors scoring at least 80% in test assessing their knowledge of counselling cards and protocol.

♦ *Coverage and timing of CBSV visits: *Detailed information concerning CBSV visits is collected on a PROCESS form administered to a random subsample of 200-300 recently delivered women each month. Indicators include: % recently delivered women who received full complement of 5 home visits; % visited according to schedule; % who received ante-natal visits; % who received post-natal visits; % who received first postnatal visit within 24 hours after delivery.

♦ *Quality of CBSV visits: *This will be assessed using the detailed DOS reporting forms completed by the supervisors during their observations of home visits, supplemented by information collected on the PROCESS form. The % CBSVs delivering the intervention according to protocol will be reported for the following: counselling cards & interactions; weighing & assessment for danger signs; referral & care seeking; correct card filling.

In-depth interviews and focus group discussions will also be carried out with a range of respondents (recently-delivered women, their families, CBSVs, TBAs, health facility staff) to explore all aspects of the intervention delivery and response to recommendations. Special sub-studies will focus on the provision of special care for low birthweight babies, and the assessment and referral of sick and very low birthweight babies.

Summary statistics and graphs showing trends over time will be compiled for all the process indicators, and determinants of quality of intervention delivery explored. The transcripts from in-depth interviews and focus group discussions will be formally analyzed using Nvivo software. Key analytical categories will be identified and the interviews systematically indexed into these categories and interpreted in order to make recommendations concerning intervention implementation, identify factors contributing to success, document barriers encountered and strategies adopted to tackle them, and identify issues important for scaling up.

### Cost and cost-effectiveness evaluation

A detailed costing of the development, set-up, and implementation of the Newhints intervention is being carried out with the following objectives: to estimate cost per life saved, if Newhints successfully reduces neonatal mortality; to estimate the incremental cost-effectiveness of Newhints relative to current practice, and compared with other newborn health interventions (in Ghana and elsewhere); to evaluate the financial sustainability (measured in terms of incremental budget implications) of the programme for the DHMTs; and to model the costs of scaling-up to regional/national levels. Both financial and economic costs will be considered. Formative research costs will be included as programme development costs; however, all other research costs will be excluded. A provider perspective will be taken and costs up to district level will be included.

Financial cost data will be collected from a variety of sources including itemized project accounting records, activity diaries, and semi-structured interviews and time sheets to determine the time allocation of Newhints team members between research and programme activities. The incremental costs of increased health facility utilization attributable to the intervention will be estimated by combining utilization data from the BIRTH and INFANT forms with data extracted from hospital records and direct observation in health centres on the quantities of drugs and supplies used for deliveries and newborn admissions, and unit cost data obtained from hospital pharmacists and regional medical stores. The economic cost of CBSV time will be quantified using information on the number and average duration of CBSV visits and other Newhints activities per month extracted from CBSV records, DOS and PROCESS forms, while in-depth interviews with CBSVs will explore the opportunity cost of this time, including possible seasonal variations.

### Informed consent

Informed consent was sought in late 2007 from all women of reproductive age living in the intervention and control zones for permission to use their surveillance data for the evaluation of NewHints, in addition to its use for the ObaapaVitA trial. Resident surveillance fieldworkers read an information sheet and consent form to the women in their own local language and checked for understanding before requesting consent. Agreement was indicated by signature or other imprint on prepared consent forms. Women were assured of their right to refuse consent without prejudice to their position in the ongoing ObaapaVitA trial (which finished in October 2008), or to any community or health services received. There were no refusals. This consent procedure is being applied on an ongoing basis for new women who move into the trial area and are recruited into the surveillance system. In addition, in the intervention zones, the CBSVs will, as per usual practice, obtain permission to make home visits from each pregnant woman identified.

Individual informed consent is also being sought from those selected for in-depth interviews and focus group discussions as part of the process and cost evaluations, and will follow a similar procedure. Interviewers read an information sheet and consent form to potential participants in their own local language and check for understanding before consent is requested. Agreement to participate in the interview is indicated by signature or other imprint on prepared consent forms. The individual's right to refuse consent or to stop the interview at any time after consent has been given will be preserved without prejudice to their position in other ongoing research, or to any community or health services received. They will not be required to provide explanation for such decisions.

No informed consent is being obtained from the DiPS or the CBSVs regarding collection of routine data from workbooks to monitor progress, or for recording observation of home visits, since such monitoring is an integral part of normal supervision activities, necessary to ensure the integrity of the intervention.

Confidentiality of all data collected is maintained at all times and is accessible only to senior project staff and to the trial monitoring committees. This includes information collected during the process evaluation except where it relates to routine monitoring of performance of CBSVs and supervisors. All women and babies in the surveillance database are identified by a unique ID number. The database is stored on a security protected server, with password access only by senior project staff. The data forms are stored in secure record stores and will be kept for a minimum of 5 years after the end of the trial.

### Trial monitoring

The Trial Steering Committee (TSC) has 12 external members, chosen to facilitate dissemination and uptake of any findings within Ghana as well as to provide technical support; members include key policy makers from the Ghana Health Service at national and regional level, national WHO and UNICEF representatives and advisers with expertise in obstetrics, demography, statistical methods, clinical trials and health services research. It is also attended by the principle investigators, members of the trial management team and representatives from the participating DHMTs and funding bodies. The Data Monitoring and Ethics Committee (DMEC) has five members, with expertise in epidemiology and medical statistics (including the design and analysis of cluster randomised trials), obstetrics, maternal health and community medicine. Both committees meet annually to examine trial conduct and progress and to advise the trial management team. The DMEC are not carrying out any interim analyses, as the Newhints intervention is health promoting and does not involve any drugs or medical procedures, and as the evaluation is based on births occurring over a period of just one year.

### Ethical approval

The trial protocol was reviewed and approved by the ethics committees of the Ghana Health Services, the Kintampo Health Research Centre and the London School of Hygiene and Tropical Medicine. It is registered with clinicaltrials.gov (identifier NCT00623337).

### Dissemination of Trial Findings

Trial findings will be shared promptly with the Technical Steering Committee, and discussed with the local District Health Management Teams. Local dissemination meetings with the study populations will be held. A CD will be compiled containing all intervention materials plus a detailed implementation evaluation report of lessons learned and shared widely. Policy briefs will be prepared and circulated nationally and internationally to relevant policy and donor organisations, and if possible a national workshop held to discuss the findings, lessons learnt concerning implementation and policy implications.

Trial findings will also be disseminated in scientific meetings and papers on: the impact of the intervention on neonatal mortality; impact on neonatal care practices; any intervention differences by place of delivery or between rural and urban zones; process outcomes, and lessons learned concerning working with volunteers, supervision, monitoring performance; training volunteers to assess babies and how well do they do; strategies to promote coverage; factors influencing response to specific care recommendations including special care for low birthweight babies and referrals; and cost-effectiveness of the intervention.

Requests to analyse or publish data from persons external to the study will be entertained 3 years after the databases are frozen. The requesting researcher in addition to at least 2 persons from within the project team will author such publications and acknowledgement will be given to the project team including the collaborators.

## Competing interests

The authors declare that they have no competing interests.

## Authors' contributions

The paper was drafted by BRK; all authors reviewed the paper, approved the final manuscript, and had major inputs to the trial design and intervention development. BRK, AM, CTA, SOA, ZH were responsible for the overall design; AM, ZH, BW, TG, SOA for DHMT coordination; AM, SD, BW, TG for mapping zones; ZH, CTA, AM, GA, BRK for development of the home visits content; AM, ZH, BRK, BW, TG, SS, GA for the training and supervision materials; ZH, EL, AM, TG, BW, GA, for sensitisation protocols; ZH, CTA, AM, BW, TG, SS, SD, BRK, GA for data collection instruments; SD, SS, BRK, GA for database design and management; BRK, AM, CTA and ZH for the analysis plan; and CP, KH for design of the cost and cost-effectiveness evaluation.
